# Editorial: Closure of abdominal wall - status Quo

**DOI:** 10.3389/jaws.2026.16896

**Published:** 2026-05-20

**Authors:** René H. Fortelny, Nadia A. Henriksen

**Affiliations:** 1 Medical Faculty, Sigmund Freud Private University, Vienna, Austria; 2 Digestive Disease Center, Bispebjerg Hospital, Copenhagen, Denmark

**Keywords:** AW-closure, hernia prevention, laparotomy, midline closure, small bites

Incisional hernia (IH) remains one of the most frequent complications following midline laparotomy, with an overall prevalence of approximately 10% and markedly higher rates in high-risk populations. Despite advances in surgical technique, prevention strategies are not uniformly implemented, particularly in emergency settings where patient-related risk factors are compounded.

Recent evidence highlights the importance of risk stratification. Surgical site infection and reoperation represent the most significant contributors to IH development, supporting a targeted and individualized preventive approach. Prophylactic mesh reinforcement has consistently demonstrated a reduction in IH incidence across meta-analyses; however, its clinical adoption remains limited due to increased risks of seroma and surgical site infection, as well as heterogeneity in study design and patient populations. Notably, randomized data in emergency laparotomy settings remain inconclusive, emphasizing the need for further investigation in high-risk cohorts.

Technical aspects of abdominal wall closure continue to play a central role. The small-stitch technique and meticulous tissue handling are key principles, with emerging technologies suggesting potential benefits in reducing tissue trauma and standardizing closure. In complex abdominal wall reconstruction, innovative approaches such as intraoperative fascial traction may facilitate high rates of primary fascial closure while reducing the need for extensive component separation.

Overall, a multimodal and risk-adapted strategy integrating optimized closure techniques, selective mesh reinforcement, and advanced reconstructive concepts appears essential. Future research should focus on high-quality randomized trials, standardized methodologies, and patient-centered outcomes to refine prevention strategies and improve clinical implementation.

